# Comparison of Thigh-Based versus Groin-Based versus Lateral-Thoracic-Based Flaps for Hand Resurfacing: A Review Article

**DOI:** 10.29252/wjps.10.3.3

**Published:** 2021-09

**Authors:** Harry Whitehouse

**Affiliations:** 1 Queen Mary University London, Mile End Rd, Bethnal Green, London E1 4NS

**Keywords:** Hand, Trauma, Resurfacing, Microsurgery, Flap

## Abstract

**BACKGROUND:**

Thigh-based, groin-based and lateral-thoracic-based flaps are available for microsurgical hand resurfacing – which is the best?

**METHODS:**

BestBETS methodology was used to systematically evaluate the advantages and disadvantages. PubMed, EMBASE and Cochrane databases were searched up until Sep 2020, using the search strategy: hand re-surfacing, free-flap, groin-flap, thigh-flap, lateral thoracic-flap, advantages, and disadvantages.

**RESULTS:**

Overall, 31 papers were identified which were used to synthesize the discussion and conclusions.

**CONCLUSION:**

Thigh-based anterolateral thigh (ALT) flaps offer the greatest versatility.

## INTRODUCTION

The hand is a highly complex and specialized tool used for manipulation which is prone to injury. Resurfacing poses a unique challenge to the reconstructive surgeon; the first aim is to provide soft tissue coverage followed by restoration of motor and sensate function and aesthetics^1^. PRISMA methodology was used to systematically evaluate the advantages and disadvantages of using thigh-based, groin-based and lateral thoracic-based flaps to identify the ideal flap for hand resurfacing.

## METHODS

A literature search was performed with the following strategy: 

Population: undergoing hand resurfacing,

Intervention: thigh-based versus groin-based versus lateral thoracic-based flap,

Comparison: to one another,

Outcome: advantages and disadvantages,

Exclusion criteria: not in English language.

PubMed, EMBASE and Cochrane databases were searched up until Sep 2020, according to PRISMA guidelines, using the search strategy: hand re-surfacing, free-flap, groin-flap, thigh-flap, lateral thoracic-flap, advantages, and disadvantages. 

## RESULTS

This search strategy identified 31 papers considered relevant and are included in the discussion.

## DISCUSSION

In smaller soft tissue defects local flaps can be used however in larger or circumferential defects or those involving multiple digits a distant pedicled or free-flap may be required. Free tissue transfer is now considered by many as best practice^2^.

There has been a paradigm shift in reconstructive surgery to avoid multiple-stages whilst attaining good coverage, functionality i.e. protective sensation and good range of movement, aesthetics and minimizing donor site morbidity^3^. Classically, resurfacing would involve multi stage-surgery with reconstruction and subsequent flap thinning^4^. By avoiding this, scarring, adhesion and granulation tissue development is avoided allowing good functional outcomes^5^.

The flap that fulfils all these criteria is the ‘ideal flap’ for hand resurfacing be it groin, thigh or lateral-thoracic based. Ultimately, the flap used will be determined by surgeon preference and experience considering the site and size of the defect and the ‘functional aesthetic-units and sub-units’ of the hand^6^.

For resurfacing the dorsal skin of the hand the flap should be thin, broad and pliable and able to stretch whilst permitting tendon gliding^1^^,^^7^. This can be achieved by thinning fascio-cutaneous flaps - despite this however they often appear bulky on the dorsum of the hand resulting in poor aesthetics and reduced function. Moreover, they often require secondary thinning which can damage the pedicle leading to skin necrosis. This can be overcome by performing a single-staged cutaneous flap thinned intra-operatively, however peri-pedicle thinning is usually avoided leaving an irregular contour. Another alternative, which is becoming popular, is ultra-thin free fascial flaps which can be surfaced with a split thickness skin-graft (SSG).

Conversely for volar skin resurfacing - a thick, glabrous, sensate surface is required which is most often provided by a fascio-cutaneous flap^1^.Sensory areas can be described as primary i.e. pulps of fingers and thumb and secondary i.e. thenar and hypothenar eminence in decreasing order of restorative importance^8^. For pulp resurfacing glabrous padded sensate tissue is required^1^. For fingers a free-fascial flap is indicated to allow tendon gliding and good range of movement^1^.

***Groin based flaps***

The groin-flap is based on the superficial circumflex iliac artery (SCIA) and has a pedicle length of 2 cm and a diameter of 1.5 mm and can be made sensate with incorporation of the lateral cutaneous nerve of intercostal nerve 12 and sartorius muscle can be incorporated for motor function. In summary advantages include a good arterial and venous supply and a concealed donor site. Disadvantages include a short pedicle, often unreliable anatomy and the flap requires thinning^9^.

The pedicled groin-flap, first described in 1972, was for many years the workhorse flap for hand resurfacing providing coverage for large hand defects^10^^,^^11^. However, in time it became unpopular for several reasons; it is a bulky flap with unreliable vascular anatomy requiring multi-staged procedures with donor site morbidity related to lymphedema including poor wound healing, seroma formation, infection, skin necrosis and persistent sensation loss. It requires a prolonged inpatient stay before pedicle division with prolonged immobilization resulting in shoulder pain and stiffness^12^. A 49 patient case-series found patients required on average 4.6 operations, with a mean hospital stay of 29+/-13 days^13^.

Pedicled groin-flaps are still sometimes indicated in children with complex injury, to cover digital stumps prior to toe transfer, high-voltage burns with a hand perfused on collateral vessels, mutilating injury as well as simultaneous defects in the fingers, hand and forearm where free flaps are not possible^14^^,^^15^. 

The free SCIA groin-flap has gained popularity with the advent of free flap surgery. It can be harvested to include the lateral femoral cutaneous nerve which is known as an extended groin-flap. It can be performed by less experienced surgeons with shorter operating times e.g. in the emergency setting. It has reliable vascular pedicle anatomy, a large skin paddle and is hairless with the scar hidden. Unlike the pedicled groin-flap early wrist physiotherapy can be started. However, like the groin flap it is bulky requiring subsequent flap thinning^3^.

Tare et al. present the free ‘mini’ groin-flap, based on the SCIA, to resurface dorsal and circumferential defects of the digits and palmar defects^2^. It has several advantages: a two-team approach reduces operating times (average 2.45 hours) and it possesses a good length pedicle with a large, hairless, skin paddle capable of covering multiple digits. The flap is primarily thinned on table to the subdermal fat layer prior to division providing a pliable flap with good cosmesis avoiding debulking procedures with the donor site closed directly. There are however several disadvantages: anatomical variation in the SCIA has been described making dissection troublesome. Multiple debulking procedures may still be required, the skin paddle is non-glabrous with poor color and texture match to the recipient site and the donor site can heal with wound stretching^2^.

Riesel et al. described the use of superficial circumflex iliac perforator (SCIP) flap for resurfacing the volar aspect of the thumb^16^. The SCIP-flap is thin and versatile providing volar coverage of the thumb whilst minimizing donor site morbidity. The underlying neurovascular structures were intact, and the SCIP-flap resurfaced the defect allowing for light touch perception restoration. Some surgeons however avoid the SCIP flap due to ‘anatomical variability’ however a study found that the medial perforator originates from the SCIA 94% of the time^17^.

A series of SCIP-flap reconstructions were presented for moderate sized upper-limb defects^4^. Praising it as a thin flap thereby avoiding thinning procedures which allows for good function and aesthetics^2^. It is raised in the ‘supra-scarpa’ plane and is considered the thinnest flap to date. It can be quickly and reliably raised with the concealed donor site healing well. They describe it as a potential workhorse flap. Advantages are similar to the groin flap additionally the time to elevate can be quick as a deep dissection is avoided. The disadvantages of the SCIP-flap include a small sometimes hypoplastic vessel requiring super microsurgery, a short pedicle and a moderately sized skin paddle^2^^,^^9^.

***Thigh based flaps***

The anterolateral thigh flap (ALT) is based on the descending branch of the lateral circumflex femoral artery. It has a long pedicle of 12 cm and vessel diameter of 2.1 mm. The lateral femoral cutaneous nerve can be coapted providing a sensate flap with vastus transferred for motor function. Advantages: versatile and easily raised with a long pedicle. Disadvantages: it is hairy, color mismatch can occur and SSGs are sometimes required at the donor site^9^.

Adani et al. presented a series in which the hand was reconstructed with a thin ALT-flap^18^. Using the flap they reconstructed defects of the dorsum and palm, allowing for bone and tendon coverage, as well as the first web space following contracture release of thumb with the ALT-flap providing good width, depth and color match^19^. As aforementioned advantages include its long pedicle and good vessel and large skin paddle with low donor site morbidity. Donor sites were closed directly if the defect was less than 8cm in width with inconspicuous scarring. SSGs were at times needed and in anticipation of a large defect tissue expansion was suggested^1^. 

Flaps were thinned to 3-4mm in-situ prior to transfer with coaption of the lateral femoral cutaneous nerve. No intra or post-operative complications were reported. They utilized a two-team approach with no need for position change therefore allowing shortened operating times. Disadvantages noted included a hairy flap in men however this can be removed by laser^20^. Flap thinning is usually required which can result in skin necrosis^21^. Perforator variability can be managed prior to surgery with Doppler with a large series showing most patients have an adequate sized perforator^19^. They described a steep learning curve due to difficult intra-muscular dissection. 

A fascia-only ALT-flap can provide a thin pliable flap which can be covered with a SSG^22^^,^^23^. Again it has a long pedicle, is easily dissected and has reliable vascular anatomy. In photographic comparison of fascia-only and fascio-cutaneous flaps the fascia-only flap was voted as superior noting less atrophy, no need for flap thinning or contouring procedures with no SSG required for coverage of donor site^22^.

***Lateral Thoracic based flaps***

Finger defects can be resurfaced with thoracodorsal artery perforator-flaps based on septo-cutaneous perforators from the thoracodorsal artery^3^. Advantages include its large hairless skin paddle and long pedicle of 10 cm with a diameter of 1mm with minimal donor site morbidity. It is muscle sparing and can be harvested in a supine position as a chimeric flap for complex reconstructions involving large soft tissue defects. Coaption of intercostal nerves allows for sensory restoration.

Disadvantages include a technically challenging flap harvest with complex variability in nervous supply posing challenges and long harvests of a sensate flap^24^. Subsequent essential thinning of the flap, containing a separated perforator and nerve, can damage intercostal nerves and therefore a conjoint relation between perforator and nerve is favored^3^. If a conjoint relationship is not identified then flap thinning is required in a second stage with sensory risks. In females the use of the flap can cause breast distortion^9^. 

Kim et al. described resurfacing hands and digits using a super thin (5-7 mm) latissimus dorsi perforator free-flap based on musculocutaneous perforators^25^. This has a long 12cm pedicle, allowing for good control, with a diameter of 1mm9. Exclusion of the deep adipose layer allows resurfacing of moderate to large, multi-digit and circumferential defects^26^. Patients required no further thinning or contouring surgery. They were able to raise large skin paddles with the donor site closed primarily if 10cm or less. Flap raising can be tough due to difficulties identifying the reliable perforator. Advantages and disadvantages as per the thoracodorsal artery perforator-flap.

Free serratus anterior has been used for resurfacing defects in the dorsal and palmar metacarpal surfaces^27^^,^^28^. It is a thin and pliable with ‘mobile’ connective tissue facilitating tendon gliding which is then covered with SSG. It has a long and controllable pedicle of 15 cm. Brody et al. utilized the three inferior slips which were then divided allowing for contouring^29^. They note difficulty raising related to avoiding the long thoracic nerve to prevent scapula-winging. Serratus muscle undergoes subsequent atrophy and the flap is therefore not as bulky as a fascio-cutaneous flap with patients regaining function and a smooth contour. There is minimal donor site morbidity and two teams can simultaneously work. Pre-disposition to venous congestion has been described therefore excessive compression is avoided to minimize necrosis. 

***Combination flaps***

The literature describes use of flaps in combination with others. Predominantly ALT with free-toe transfer and combined groin-ALT pedicled flaps to create a sandwich^30^^-^^33^. 

Kim et al. resurfaced a totally degloved hand using an ALT-flap for palmar resurfacing and a paraumbilical perforator-flap for the dorsal resurfacing^34^. They incorporated branches of the lateral femoral cutaneous nerve and thinned the flap to 7 mm. In combination a mitten was formed later requiring interdigitation and thinning. At 18 months the patient had good manual dexterity and could discriminate 10mm of static two-point discrimination. The ALT has a large skin paddle, long pedicle and minimal donor site morbidity. If perforators cannot be identified then a pedicled ALT can be used^35^^,^^36^.

Moreover, in a study combined multi-lobed flaps were used^5^. This paper discusses and presents one-staged reconstruction of complex soft tissue injuries in the hands using ‘fabricated or multi-lobed ALT chimeric free-flaps’ with lateral femoral nerve coaption. ALT chimeric multi-lobed flaps necessitate only one anastomosis with several flaps offering a plethora of designs to fill the defect. However, this is advanced microsurgery which should be performed by experienced surgeons and can leave patients with large scars.

## CONCLUSION

Groin based, thigh based and lateral thoracic based flaps all have their relative advantages and disadvantages. Every hand injury is different, and many factors must be considered prior to resurfacing. The ideal flap for hand resurfacing is the thigh-based ALT-flap; a large super-thin fascial free-flap can be raised and overlay with an SSG providing aesthetically pleasing dorsal-hand and finger resurfacing with good function with avoidance of subsequent thinning procedures. Similarly, a large, hairless (sometimes requiring lasering), fascio-cutaneous sensate fap can be raised and thinned to the desired thickness for 1^st^ web-space reconstruction or resurfacing of volar defects. It has a long reliable, good diameter pedicle with coaption of nerve and inclusion of muscle possible. The donor site heals well requiring SSG at times which can be overcome with pre-operative tissue expansion. Similarly, pre-operative Doppler may be indicated. Multi-lobed ALT chimeric free-flaps open the door to infinite possibility however these should only be attempted in centres with high expertise.

## CONFLICT OF INTEREST

I hereby declare that there are no conflicts of interest
